# High Tumor Infiltrating Lymphocytes Are Associated with Overall Survival and Good Prognostic Parameters in Endometrial Endometrioid Carcinoma Patients

**DOI:** 10.5146/tjpath.2022.01596

**Published:** 2023-01-15

**Authors:** Cigdem Ozturk, Gokce Askan, Seda Duman Ozturk, Oguzhan Okcu, Bayram Sen, Recep Bedir

**Affiliations:** Recep Tayyip Erdogan University Training and Research Hospital, Department of Pathology, Rize, Turkiye; Recep Tayyip Erdogan University, Faculty of Medicine, Department of Pathology, Rize, Turkiye; Recep Tayyip Erdogan University Training and Research Hospital, Department of Biochemistry, Rize, Turkiye

**Keywords:** Tumor-infiltrating lymphocytes, Endometrial carcinomas, Prognosis, Practical parameter

## Abstract

***Objective:*** The mortality incidence of endometrial carcinomas (ECs) has increased in recent years. Therefore, recent studies have focused on the cellular and microenvironmental properties of ECs. Tumor-infiltrating lymphocytes (TILs), a component of the microenvironment, have been found to be associated with the prognosis in many tumors. Although TILs were mostly evaluated by immunohistochemical studies in ECs, in our study, the evaluation was done with a light microscope as a practical approach, and we aimed to determine the prognostic importance of TILs in endometrioid ECs.

***Material and Method:*** 104 patients were included in the study. TILs in the stromal area (sTILs) were evaluated on hematoxylin and eosin (HE) stained-sections at X200 objective. The presence of TILs was evaluated as follows; 0-10% as low, 20-40% as moderate, and 50-90% as intense. Then TILs were grouped as low and high.

***Results:*** Tumors with high TILs were more prone to have FIGO (International Federation of Gynecology and Obstetrics) grade 1 tumors, low nuclear grade, early pathological stage, smaller size, no lymphovascular invasion, myometrial invasion below 50%, and no cervical involvement. In the presence of high TILs, the overall survival showed significant increase but no significant correlation was found with disease-free survival.

***Conclusion:*** Interest in the molecular properties of ECs has increased in recent years. TIL, which can be easily evaluated in HE sections, is an important parameter in patient selection for molecular tests and determining the prognosis of patients.

## INTRODUCTION

Endometrial carcinomas (ECs) are the most common gynecological malignancies in developed countries (1,2). Due to rise in the mortality incidence of EC, recent studies have focused on determining the risk by evaluating the histopathological features (1–3).

Endometrioid type endometrial carcinoma (ECC) was divided into 4 different molecular subtypes in 2013 (4). These subtypes are *POLE* ultramutated, microsatellite instable (hypermutated), copy number low, and copy number high (4). In the following years, many validations and recommendation studies have been carried out on these classifications (5).

Cellular properties of the tumor as well as the tumor microenvironment play a role in tumor progression and metastasis (6). Various studies have been conducted to determine the immune response components of the microenvironment in ECCs by immunohistochemical methods. The majority of these studies are based on the association between immune response and molecular subtypes. This is because molecular testing is technically difficult, not always readily available, and has a financial burden. Therefore, it seems that determining the component of the microenvironment by immunohistochemistry is more cost-effective (7–9). However, it is not always possible to conduct immunohistochemistry in daily pathology practice, and not practical to evaluate several immunohistochemical markers separately.

In 2017, the International Tumor-Infiltrating Lymphocytes Working Group (ITILWG) standardized the evaluation of TILs in various solid tumors, based on the methodology in breast cancers (10,11). Accordingly, TIL is evaluated only under a light microscope, which is easy to apply and much more practical. In our study, with the recommendation of ITILWG, we aimed to determine the prognostic importance of TILs in ECCs, regardless of molecular subtypes.

## MATERIAL and METHODS

### Patients and Tissue Samples

Patients who underwent hysterectomy for ECC at our hospital between January 2010 and December 2020 were included in our study. Hematoxylin and eosin (HE) stained-sections were retrieved from the pathology archive. Patients whose paraffin-embedded blocks and/or HE stained-slides were not obtained, who had insufficient clinical information, who underwent neoadjuvant radiotherapy or distant organ metastasis at the time of diagnosis, as well as those with a second primary or who died within the first month after surgery were excluded from the study. As a result, a total of 104 patients with ECC were included.

### Patients’ Characteristics

The age, adjuvant treatment history, status of metastasis, and survival data were obtained from the hospital database. Tumor size information was obtained from the pathology reports, while FIGO (International Federation of Gynecology and Obstetrics) grade, nuclear grade, pathological stage, myometrium invasion rate, lymphovascular invasion, lymph node metastasis, and necrosis were re-evaluated. All were classified as endometrioid type endometrial adenocarcinoma according to the World Health Organization (WHO) criteria and were graded according to FIGO (12,13).

### Evaluation of Tumor-Infiltrating Lymphocytes (TILs)

In our study, TILs in the tumor stroma were evaluated, based on the recommendation of the ITILWG, since it is easier, more reliable, and reproducible than the intratumoral area (10,11). The assessment was done within the invasive component of the tumor, while areas of endometrial hyperplasia or necrosis around the tumor were not evaluated. It has been recommended to evaluate TIL in all solid tumors by HE stained-sections under a light microscope with a x200 or x400 objective.

**Figure 1:**
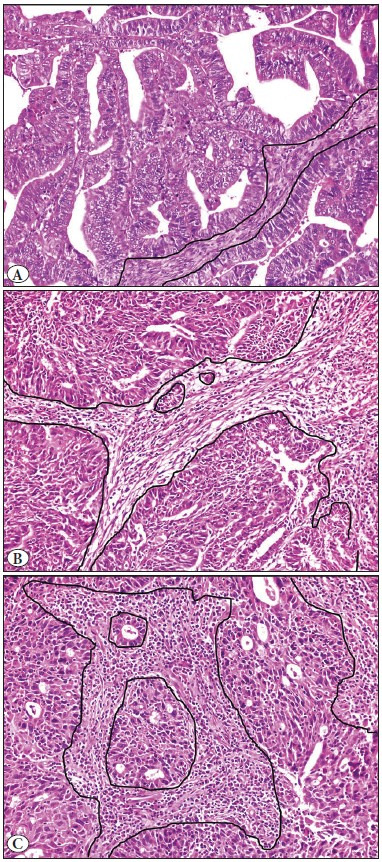
Hematoxylin and eosin stained 4 μm sections of endometrioid type endometrial carcinoma. **A)** Example of low Tumor infiltrating Lymphocytes (H&E x400), **B)** moderate Tumor infiltrating Lymphocytes (H&E x200), **C)** Intense Tumor infiltrating Lymphocytes patients (H&E x200).

In our study, TILs were evaluated by three pathologists (CO, GA, OO) under the x200 objective (Olympus, BX-51, ocular 22 mm, field size 0.950 mm2) blinded to the clinical and other pathological features of the patients. The patients with different scores were re-evaluated under a five-headed microscope and consensus was achieved. The presence of TIL was evaluated as follows: 0-10% low, 20-40% moderate, and 50-90% intense (Figure 1). The low and moderate groups were taken as a single group, since the number of cases forming the groups was low. As a result, the TIL groups were divided into two as low and high.

### Ethical Approval

Ethics committee confirmation for our study was obtained from the non-interventional clinical research ethics committee of Recep Tayyip Erdogan University Faculty of Medicine (E-40465587-050.01.04-439). The study was conducted under the Declaration of Helsinki and the ethical standards of the institutional research committee and the Reporting recommendations for tumor marker prognostic studies (REMARK) guidelines (14).

### Statistical Analysis

Statistical analyses were performed using IBM SPSS Statistics, Version 22.0 (SPSS Inc., Chicago, USA). Fleiss Kappa analysis was used to compare the agreement of TIL assessment among three pathologists. The Chi-square test (Pearson Chi-square) or Fisher’s Exact test, where appropriate, was used to compare the correlation of categorical variables. The possible factors identified with univariate analyses were further entered into the Cox regression analysis with backward selection to specify independent predictors of overall and disease-free survival and a hazard ratio (HR) with 95% CI is presented. The Kaplan-Meier method was used for survival analysis and evaluation was performed with the log-rank test to identify the effect of tumor infiltrating lymphocytes on survival. For statistical significance, a 5% type-1 error level was used.

## RESULTS

### General Characteristics

The mean age of the 104 patients included in the study was 59 years (ranging from 41 to 87). The majority of the patients (76%) were in the early stage at the time of diagnosis. 61.5% of the patients were FIGO grade 1, 33% were FIGO grade 2, and 5.8% were FIGO grade 3. Lymph node dissection was performed in 83 (80%) patients, and lymph node metastasis was observed in 6 (6%). The clinicopathological characteristics of the patients are summarized in Table 1.

**Table 1:** Clinicopathological characteristics of the patients.

		**n**	**%**
Tumor Infiltrating Lymphocytes	0-10%	21	(20)
	20-40%	31	(30)
	50-90%	52	(50)
FIGO grade	1	64	(61.5)
	2	34	(33)
	3	6	(6)
Nuclear grade	1	27	(26)
	2	70	(67)
	3	7	(7)
Pathologic stage	1a	79	(76)
	1b	15	(14)
	2	10	(10)
Tumor size	< 3 cm	53	(51)
	≥ 3 cm	51	(49)
Lymphovascular invasion	Negative	82	(79)
	Positive	22	(22)
Myometrial invasion ratio	<50	84	(81)
	≥50	20	(19)
Necrosis	Negative	85	(82)
	Positive	19	(18)
Cervical invasion	Negative	94	(90)
	Positive	10	(10)
Lymph node status	Negative	77	(93)
	Positive	6	(7)
Adjuvant chemotherapy	Negative	89	(86)
	Positive	15	(14)
Distant metastasis	Negative	94	(90)
	Positive	10	(10)
Survival status	Alive with/no disease	94	(90)
	Death of disease	10	(10)

### Relationship Between TIL and Clinicopathological Parameters

The Fleiss kappa coefficient revealed an almost perfect agreement in TILs assesment (kappa value: 0.938 (p< 0.001)) among the three observers (CO, GA and OO). The majority of patients with high TILs were FIGO grade 1 (p=0.016). Patients with high TILs were more prone to have low nuclear grade (p=0.001). A high TILs value was also associated with early pathological stage, smaller tumor size, myometrial invasion below 50%, no lymphovascular invasion, and no cervical involvement. The relationship between TILs and the clinicopathological parameters is shown in Table 2.

**Table 2:** Clinicopathological parameters and TILs.

		**Tumor Infiltrating Lymphocytes**				
		**0-40%**		**50-90%**		
		**n**	**(%)**	**n**	**(%)**	**p value**
FIGO grade	1	26	(50)	38	(73)	0.016
	2+3	26	(50)	14	(27)	
Nuclear grade	1	6	(11.5)	21	(40)	0.001
	2+3	46	(88.5)	31	(60)	
Pathologic stage	1a	33	(63.5)	46	(88.5)	0.011
	1b	11	(21)	4	(8)	
	2	8	(15)	2	(4)	
Pathologic stage	1a	33	(63.5)	46	(88.5)	0.003
	1b+2	19	(36.5)	6	(11.5)	
Tumor size	< 3 cm	20	(38.5)	33	(63.5)	0.011
	≥3 cm	32	(61.5)	19	(36.5)	
Lymphovascular invasion	Negative	37	(71)	45	(86.5)	0.055
	Positive	15	(29)	7	(13.5)	
Myometrial invasion ratio	<50	37	(71)	47	(90)	0.013
	≥50	15	(29)	5	(10)	
Necrosis	Negative	40	(77)	45	(86.5)	0.205
	Positive	12	(23)	7	(13.5)	
Cervical invasion	Negative	44	(85)	50	(96)	0.046
	Positive	8	(15)	2	(4)	
Lymph node status	Negative	37	(90)	40	(95)	0.433
	Positive	4	(10)	2	(5)	
Adjuvant chemotherapy	Negative	45	(86.5)	44	(85)	0.78
	Positive	7	(13.5)	8	(15)	
Distant metastasis	Negative	47	(90)	47	(90)	1.000
	Positive	5	(10)	5	(10)	
Survival status	Alive with/no disease	44	(85)	50	(96)	0.046
	Death of disease	8	(15)	2	(4)	

### TILs and Prognostic Association with Outcome

**Figure 2:**
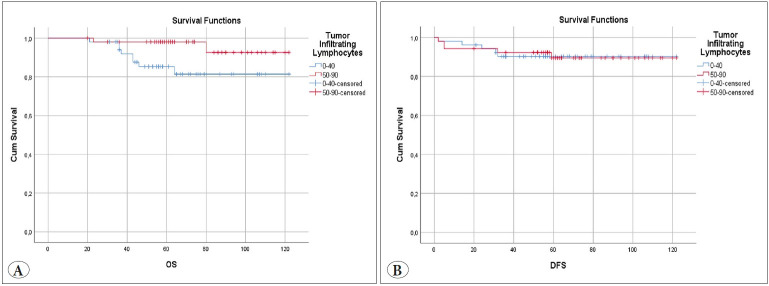
**A)** Kaplan Meier overall survival curve Tumor infiltrating Lymphocytes (p=0.035), **B)** Kaplan Meier disease free survival curves for Tumor infiltrating Lymphocytes (p=0.952).

The median follow up of patients was 62 months, ranging from 20 to 122 months. Ten (10%) died during the follow-up and distant organ metastases developed in 10 (10%) patients. In the presence of high TILs, the overall survival increased statistically (p=0.035). No significant correlation was found with disease-free survival (p=0.952). Kaplan Meier curves of the patients are shown in Figure 2. In Cox regression analysis, TILs (Hazard ratio (HR) 0.218; 95% CI 0.146-1.032; p: 0.055), age, FIGO grade, and lymphovascular invasion were found to be risk factors for OS, in univariate analysis. However, in multivariate analysis, TILs were not an independent prognostic variable for OS (Table 3). For DFS, TILs were not an independent prognostic variable in univariate and multivariate analyzes.

**Table 3:** Univariate and multivariate analysis results for overall survival.

	**Univariate**		**Multivariate**	
	**p**	**HR (95% CI)**	**p**	**HR (95% CI)**
Tumor Infiltrating Lymphocytes	0.055	0.218 (0.046-1.032)		
FIGO grade	0.01	15.209 (1.922-120.378)	0.015	13.078 (1.631-104.851
Nuclear grade	0.243	33.526 (0.092-12216.16)		
Pathologic stage	0.323	1.893 (0.533-6.72)		
Tumor size	0.598	1.406 (0.396-4.998)		
Lymphovascular invasion	0.026	4.092 (1.183-14.152)		
Myometrial invasion ratio	0.126	2.69 (0.757-9.562)		
Necrosis	0.487	0.481 (0.061-3.795)		
Cervical invasion	0.089	3.255 (0.836-12.68)		
Distant metastasis	0.002	7.798 (2.191-27.756)		
Age	0.002	1.105 (1.037-1.177)	0.006	1.082 (1.023-1.143)

## DISCUSSION

Various immune cells such as dendritic cells, natural killer cells, mast cells, macrophages, and lymphocytes contribute to the tumor microenvironment (15). Some of these components show antitumoral activity, while some facilitate neoangiogenesis and growth of the tumor (15,16). For example, CD8 T lymphocytes, dendritic cells, tumor antigen-specific T lymphocytes, CD45 RO+ T lymphocytes, and tumor-associated memory T lymphocytes show antitumor activity. By contrast, macrophages or Fox P3 + regulatory T lymphocytes increase tumor invasion, progression, metastasis, and neoangiogenesis (16).

Most studies in ECCs have focused on the association between TILs and molecular status rather than the prognostic significance of the TILs (12). ECCs with mutations in DNA mismatch repair genes (MMR), one of the molecular subtypes, occur in approximately 10-20% of all ECCs (17). Recent studies have shown that TILs play a role in predicting MMR defects (18–20). Another molecular subtype, the *POLE* mutant type, is observed in 7% (21). TILs count was reported to be higher in this subtype compared to *POLE* wild type (21,22). There are also studies reporting that immune checkpoint inhibitors can be used in the treatment of tumors with mutations (23–25). Therefore, it is important to report TILs both in patient selection for molecular tests and in the treatment management of patients.

There are also studies mainly focused on the relationship between MMR and *POLE* status rather than the prognostic value of TILs in ECCs (26–32). de Jong et al. have found high CD8+T lymphocyte value, high CD8+/FoxP3+ ratio, and the presence of CD45 RO+ T lymphocytes to be good prognostic parameters. Moreover, they have been associated with long DFS and OS (26). On the other hand, Giatromalaki et al. have argued that an increase in FOXP3 released from CD4+ CD25+ regulator T cells (Tregs) is associated with increased neoangiogenesis and poor prognosis by suppressing effector T cells (27). Similar to this study, Yamagami et al. have associated high Tregs with poor DFS (28). Jiang et al. have investigated the presence of tumor-associated macrophages (TAMs), which are thought to have a role in tumor progression in patients with ECC and endometrial hyperplasia. According to this study, TAMs play a role in the progression of precancerous endometrial lesions to ECCs (29). In addition, several studies in the literature claim that intraepithelial CD8+ T lymphocytes are an independent prognostic factor and that stromal CD3+ T lymphocytes have a prognostic value (26,30–32). The results of studies advocating the relationship of all these immune components with the prognosis have shown that TIL is a prognostic parameter. In our study, TILs were evaluated with the method suggested by the ITILWG. According to our results, high TILs were associated with low FIGO grade, early pathological stage, low nuclear grade, low tumor diameter, myometrial invasion below 50%, no cervical involvement, and no lymphovascular invasion. When the survival of the patients was evaluated, OS was found to be longer in the presence of high TIL. The results of our study and studies advocating the relationship of all these immune components with the prognosis have shown that TIL is a prognostic parameter.

Almost all of these studies are based on immunohistochemical studies in the literature. However, immunohistochemical work-up is not easily accessible for all pathologists. Endometrial curettage sampling is relatively easy in terms of sampling compared to many other tumors, and the curettage procedure is less material-dependent. For this reason, it can be easily performed even in many centers that do not have the opportunity to perform immunohistochemical analysis. Therefore, we evaluated TILs with light microscopy in patients with ECC, according to the recommendation of the ITILWG, without additional cost and time. Inter-observer agreement in our study was almost excellent and these results suggest that similar results will be obtained by different observers. Our study is the first study of ECs using only a light microscope, without an additional immunohistochemical study, as recommended by ITILWG.

Lymph node dissection in ECs is not recommended in patients with FIGO grade 1-2, endometrioid histology, tumor size less than 2 cm, and myometrial invasion below 50% (33). In our study, lymph node dissection was performed in 80% of the patients, and lymph node involvement was observed in 8%. Similarly, it has been reported in the literature that lymph node dissection is applied to a much larger number of patients than is recommended in daily practice for surgical staging, as well as prognosis estimation, and adjuvant treatment decision (34). However, lymph node dissection may cause various complications such as bleeding, lymphedema, inflammatory infiltration, vascular and nerve damage within the short and long term after surgery (35). In our study, lymph node metastasis was not observed in the vast majority of patients who met the dissection conditions of the guidelines. These results show the necessity of additional parameters to the existing parameters in the decision of lymph node dissection. Therefore, TILs, which are associated with prognostic parameters, can be evaluated in frozen sections during surgery or in curettage materials before surgery. By this way, TILs can be used as a parameter in the selection of patients who need lymph node dissection. No significant association was found between TIL and lymph node metastasis in our study. This result could be explained by the high number of low stage tumors in our cohort. Further studies are needed on this aspect.

Based on the ITILWG’s recommendation, TILs evaluation using a light microscope has begun to take its place in the pathology reports of many cancer types, especially in colon and breast (10). Our study supports that reporting TILs in ECCs without requiring any extra time and cost will contribute to patient prognosis.

Our study consisted of patients whose molecular subtypes were unknown, since molecular testing could not be performed in our center. Therefore, an idea regarding the association between TILs and molecular subtypes could not be obtained. Based on the association between TILs and molecular subtypes, which has been proven in many studies in the literature, TILs can contribute to patient selection for molecular testing by identifying tumors suspicious for certain mutations. Similarly, it will be very useful in patient management for centers that do not have the opportunity to perform molecular analyses.

## CONCLUSION

ECCs are observed at a very high rate in women all over the world and the knowledge about their subtypes continues to increase. It is not cost effective to refer all patients to testing for molecular subtyping. For this reason, TILs, which is evaluated practically on HE sections, is important in patient selection for molecular tests and in determining the prognosis of patients.

## Conflict of Interest

Authors report no conflict of interest.

## References

[ref-1] Travaglino Antonio, Raffone Antonio, Stradella Cristina, Esposito Rosanna, Moretta Paola, Gallo Cinzia, Orlandi Giuliana, Insabato Luigi, Zullo Fulvio (2020). Impact of endometrial carcinoma histotype on the prognostic value of the TCGA molecular subgroups. Arch Gynecol Obstet.

[ref-2] Siegel Rebecca L., Miller Kimberly D., Jemal Ahmedin (2015). Cancer statistics, 2015. CA Cancer J Clin.

[ref-3] Gilks C. Blake, Oliva Esther, Soslow Robert A. (2013). Poor interobserver reproducibility in the diagnosis of high-grade endometrial carcinoma. Am J Surg Pathol.

[ref-4] Kandoth Cyriac, Schultz Nikolaus, Cherniack Andrew D., Akbani Rehan, Liu Yuexin, Shen Hui, Robertson A. Gordon, Pashtan Itai, Shen Ronglai, Benz Christopher C., Yau Christina, Laird Peter W., Ding Li, Zhang Wei, Mills Gordon B., Kucherlapati Raju, Mardis Elaine R., Levine Douglas A., Cancer Genome Atlas Research Network (2013). Integrated genomic characterization of endometrial carcinoma. Nature.

[ref-5] Travaglino Antonio, Raffone Antonio, Mollo Antonio, Borrelli Giorgio, Alfano Pasquale, Zannoni Gian Franco, Insabato Luigi, Zullo Fulvio (2020). TCGA molecular subgroups and FIGO grade in endometrial endometrioid carcinoma. Arch Gynecol Obstet.

[ref-6] Raffone Antonio, Travaglino Antonio, Raimondo Diego, Boccellino Maria Pia, Maletta Manuela, Borghese Giulia, Casadio Paolo, Insabato Luigi, Mollo Antonio, Zullo Fulvio, Seracchioli Renato (2021). Tumor-infiltrating lymphocytes and POLE mutation in endometrial carcinoma. Gynecol Oncol.

[ref-7] Talhouk Aline, Derocher Heather, Schmidt Pascal, Leung Samuel, Milne Katy, Gilks C. Blake, Anglesio Michael S., Nelson Brad H., McAlpine Jessica N. (2019). Molecular Subtype Not Immune Response Drives Outcomes in Endometrial Carcinoma. Clin Cancer Res.

[ref-8] Raffone Antonio, Travaglino Antonio, D'Antonio Antonio, De Marco Margot, Caccese Miriam, Mascolo Massimo, Insabato Luigi, Zeppa Pio, Rosati Alessandra, Mollo Antonio, Zullo Fulvio, Guida Maurizio (2020). BAG3 expression correlates with the grade of dysplasia in squamous intraepithelial lesions of the uterine cervix. Acta Obstet Gynecol Scand.

[ref-9] Travaglino Antonio, Raffone Antonio, Saccone Gabriele, Mascolo Massimo, D'Alessandro Pietro, Arduino Bruno, Mollo Antonio, Insabato Luigi, Zullo Fulvio (2019). Nuclear expression of β-catenin in endometrial hyperplasia as marker of premalignancy. APMIS.

[ref-10] Salgado R., Denkert C., Demaria S., Sirtaine N., Klauschen F., Pruneri G., Wienert S., Eynden G., Baehner F. L., Penault-Llorca F., Perez E. A., Thompson E. A., Symmans W. F., Richardson A. L., Brock J., Criscitiello C., Bailey H., Ignatiadis M., Floris G., Sparano J., Kos Z., Nielsen T., Rimm D. L., Allison K. H., Reis-Filho J. S., Loibl S., Sotiriou C., Viale G., Badve S., Adams S., Willard-Gallo K., Loi S., International TILs Working Group 2014 (2015). The evaluation of tumor-infiltrating lymphocytes (TILs) in breast cancer: recommendations by an International TILs Working Group 2014. Ann Oncol.

[ref-11] Hendry Shona, Salgado Roberto, Gevaert Thomas, Russell Prudence A., John Tom, Thapa Bibhusal, Christie Michael, Vijver Koen, Estrada M. V., Gonzalez-Ericsson Paula I., Sanders Melinda, Solomon Benjamin, Solinas Cinzia, Eynden Gert G. G. M., Allory Yves, Preusser Matthias, Hainfellner Johannes, Pruneri Giancarlo, Vingiani Andrea, Demaria Sandra, Symmans Fraser, Nuciforo Paolo, Comerma Laura, Thompson E. A., Lakhani Sunil, Kim Seong-Rim, Schnitt Stuart, Colpaert Cecile, Sotiriou Christos, Scherer Stefan J., Ignatiadis Michail, Badve Sunil, Pierce Robert H., Viale Giuseppe, Sirtaine Nicolas, Penault-Llorca Frederique, Sugie Tomohagu, Fineberg Susan, Paik Soonmyung, Srinivasan Ashok, Richardson Andrea, Wang Yihong, Chmielik Ewa, Brock Jane, Johnson Douglas B., Balko Justin, Wienert Stephan, Bossuyt Veerle, Michiels Stefan, Ternes Nils, Burchardi Nicole, Luen Stephen J., Savas Peter, Klauschen Frederick, Watson Peter H., Nelson Brad H., Criscitiello Carmen, O'Toole Sandra, Larsimont Denis, Wind Roland, Curigliano Giuseppe, André Fabrice, Lacroix-Triki Magali, Vijver Mark, Rojo Federico, Floris Giuseppe, Bedri Shahinaz, Sparano Joseph, Rimm David, Nielsen Torsten, Kos Zuzana, Hewitt Stephen, Singh Baljit, Farshid Gelareh, Loibl Sibylle, Allison Kimberly H., Tung Nadine, Adams Sylvia, Willard-Gallo Karen, Horlings Hugo M., Gandhi Leena, Moreira Andre, Hirsch Fred, Dieci Maria V., Urbanowicz Maria, Brcic Iva, Korski Konstanty, Gaire Fabien, Koeppen Hartmut, Lo Amy, Giltnane Jennifer, Rebelatto Marlon C., Steele Keith E., Zha Jiping, Emancipator Kenneth, Juco Jonathan W., Denkert Carsten, Reis-Filho Jorge, Loi Sherene, Fox Stephen B. (2017). Assessing Tumor-Infiltrating Lymphocytes in Solid Tumors: A Practical Review for Pathologists and Proposal for a Standardized Method from the International Immuno-Oncology Biomarkers Working Group: Part 2: TILs in Melanoma, Gastrointestinal Tract Carcinomas, Non-Small Cell Lung Carcinoma and Mesothelioma, Endometrial and Ovarian Carcinomas, Squamous Cell Carcinoma of the Head and Neck, Genitourinary Carcinomas, and Primary Brain Tumors. Adv Anat Pathol.

[ref-12] Matias-Guiu X, Oliva E, McCluggage WG (2020). Tumours of the uterine corpus. Female Genital Tumours.

[ref-13] Amant Frédéric, Mirza Mansoor Raza, Koskas Martin, Creutzberg Carien L. (2018). Cancer of the corpus uteri. Int J Gynaecol Obstet.

[ref-14] Sauerbrei Willi, Taube Sheila E., McShane Lisa M., Cavenagh Margaret M., Altman Douglas G. (2018). Reporting Recommendations for Tumor Marker Prognostic Studies (REMARK): An Abridged Explanation and Elaboration. J Natl Cancer Inst.

[ref-15] Soo Ross A., Chen Zhaojin, Yan Teng Rebecca Siew, Tan Hon-Lyn, Iacopetta Barry, Tai Bee Choo, Soong Richie (2018). Prognostic significance of immune cells in non-small cell lung cancer: meta-analysis. Oncotarget.

[ref-16] Guo Fang, Dong Yishan, Tan Qingqing, Kong Jing, Yu Bin (2020). Tissue Infiltrating Immune Cells as Prognostic Biomarkers in Endometrial Cancer: A Meta-Analysis. Dis Markers.

[ref-17] Salvesen H. B., MacDonald N., Ryan A., Iversen O. E., Jacobs I. J., Akslen L. A., Das S. (2000). Methylation of hMLH1 in a population-based series of endometrial carcinomas. Clin Cancer Res.

[ref-18] Hampel Heather, Frankel Wendy, Panescu Jenny, Lockman Janet, Sotamaa Kaisa, Fix Daniel, Comeras Ilene, La Jeunesse Jennifer, Nakagawa Hidewaki, Westman Judith A., Prior Thomas W., Clendenning Mark, Penzone Pamela, Lombardi Janet, Dunn Patti, Cohn David E., Copeland Larry, Eaton Lynne, Fowler Jeffrey, Lewandowski George, Vaccarello Luis, Bell Jeffrey, Reid Gary, Chapelle Albert (2006). Screening for Lynch syndrome (hereditary nonpolyposis colorectal cancer) among endometrial cancer patients. Cancer Res.

[ref-19] Mills Anne M., Longacre Teri A. (2016). Lynch Syndrome Screening in the Gynecologic Tract: Current State of the Art. Am J Surg Pathol.

[ref-20] Shia Jinru, Black Destin, Hummer Amanda J., Boyd Jeff, Soslow Robert A. (2008). Routinely assessed morphological features correlate with microsatellite instability status in endometrial cancer. Hum Pathol.

[ref-21] Church David N., Briggs Sarah E. W., Palles Claire, Domingo Enric, Kearsey Stephen J., Grimes Jonathon M., Gorman Maggie, Martin Lynn, Howarth Kimberley M., Hodgson Shirley V., Kaur Kulvinder, Taylor Jenny, Tomlinson Ian P. M., NSECG Collaborators (2013). DNA polymerase ε and δ exonuclease domain mutations in endometrial cancer. Hum Mol Genet.

[ref-22] Van Gool Inge C., Ubachs Jef E. H., Stelloo Ellen, Kroon Cor D., Goeman Jelle J., Smit Vincent T. H. B. M., Creutzberg Carien L., Bosse Tjalling (2018). Blinded histopathological characterisation of POLE exonuclease domain-mutant endometrial cancers: sheep in wolf's clothing. Histopathology.

[ref-23] Howitt Brooke E., Shukla Sachet A., Sholl Lynette M., Ritterhouse Lauren L., Watkins Jaclyn C., Rodig Scott, Stover Elizabeth, Strickland Kyle C., D'Andrea Alan D., Wu Catherine J., Matulonis Ursula A., Konstantinopoulos Panagiotis A. (2015). Association of Polymerase e-Mutated and Microsatellite-Instable Endometrial Cancers With Neoantigen Load, Number of Tumor-Infiltrating Lymphocytes, and Expression of PD-1 and PD-L1. JAMA Oncol.

[ref-24] Hussein Yaser R., Weigelt Britta, Levine Douglas A., Schoolmeester J. Kenneth, Dao Linda N., Balzer Bonnie L., Liles Georgia, Karlan Beth, Köbel Martin, Lee Cheng-Han, Soslow Robert A. (2015). Clinicopathological analysis of endometrial carcinomas harboring somatic POLE exonuclease domain mutations. Mod Pathol.

[ref-25] Gool Inge C., Eggink Florine A., Freeman-Mills Luke, Stelloo Ellen, Marchi Emanuele, Bruyn Marco, Palles Claire, Nout Remi A., Kroon Cor D., Osse Elisabeth M., Klenerman Paul, Creutzberg Carien L., Tomlinson Ian Pm, Smit Vincent Thbm, Nijman Hans W., Bosse Tjalling, Church David N. (2015). POLE Proofreading Mutations Elicit an Antitumor Immune Response in Endometrial Cancer. Clin Cancer Res.

[ref-26] Jong R. A., Leffers N., Boezen H. M., Hoor K. A., Zee A. G. J., Hollema H., Nijman H. W. (2009). Presence of tumor-infiltrating lymphocytes is an independent prognostic factor in type I and II endometrial cancer. Gynecol Oncol.

[ref-27] Giatromanolaki Alexandra, Bates Gaynor J., Koukourakis Michael I., Sivridis Efthimios, Gatter Kevin C., Harris Adrian L., Banham Alison H. (2008). The presence of tumor-infiltrating FOXP3+ lymphocytes correlates with intratumoral angiogenesis in endometrial cancer. Gynecol Oncol.

[ref-28] Yamagami Wataru, Susumu Nobuyuki, Tanaka Hideo, Hirasawa Akira, Banno Kouji, Suzuki Nao, Tsuda Hiroshi, Tsukazaki Katsumi, Aoki Daisuke (2011). Immunofluorescence-detected infiltration of CD4+FOXP3+ regulatory T cells is relevant to the prognosis of patients with endometrial cancer. Int J Gynecol Cancer.

[ref-29] Jiang Xue-feng, Tang Qiong-lan, Shen Xi-ming, Li Hai-gang, Chen Lun-hua, Wang Xiao-yu, Luo Xin, Lin Zhong-qiu, Jiang Guang-yu (2012). Tumor-associated macrophages, epidermal growth factor receptor correlated with the triple negative phenotype in endometrial endometrioid adenocarcinoma. Pathol Res Pract.

[ref-30] Kondratiev Svetlana, Sabo Edmond, Yakirevich Evgeny, Lavie Ofer, Resnick Murray B. (2004). Intratumoral CD8+ T lymphocytes as a prognostic factor of survival in endometrial carcinoma. Clin Cancer Res.

[ref-31] Čermáková Petra, Melichar Bohuslav, Tomšová Markéta, Zoul Zdeněk, Kalábová Hana, Spaček Jiří, Doležel Martin (2014). Prognostic significance of CD3+ tumor-infiltrating lymphocytes in patients with endometrial carcinoma. Anticancer Res.

[ref-32] Ino Kazuhiko, Yamamoto Eiko, Shibata Kiyosumi, Kajiyama Hiroaki, Yoshida Norio, Terauchi Mikio, Nawa Akihiro, Nagasaka Tetsuro, Takikawa Osamu, Kikkawa Fumitaka (2008). Inverse correlation between tumoral indoleamine 2,3-dioxygenase expression and tumor-infiltrating lymphocytes in endometrial cancer: its association with disease progression and survival. Clin Cancer Res.

[ref-33] Colombo N., Creutzberg C., Amant F., Bosse T., González-Martín A., Ledermann J., Marth C., Nout R., Querleu D., Mirza M. R., Sessa C., ESMO-ESGO-ESTRO Endometrial Consensus Conference Working Group (2016). ESMO-ESGO-ESTRO Consensus Conference on Endometrial Cancer: diagnosis, treatment and follow-up. Ann Oncol.

[ref-34] Vargas Roberto, Rauh-Hain J. Alejandro, Clemmer Joel, Clark Rachel M., Goodman Annekathryn, Growdon Whitfield B., Schorge John O., Del Carmen Marcela G., Horowitz Neil S., Boruta David M. (2014). Tumor size, depth of invasion, and histologic grade as prognostic factors of lymph node involvement in endometrial cancer: a SEER analysis. Gynecol Oncol.

[ref-35] How J., Gotlieb W. H., Press J. Z., Abitbol J., Pelmus M., Ferenczy A., Probst S., Gotlieb R., Brin S., Lau S. (2015). Comparing indocyanine green, technetium, and blue dye for sentinel lymph node mapping in endometrial cancer. Gynecol Oncol.

